# Dengue Virus 3 Genotype 1 Associated with Dengue Fever and Dengue Hemorrhagic Fever, Brazil

**DOI:** 10.3201/eid1402.070278

**Published:** 2008-02

**Authors:** Leandra Barcelos Figueiredo, Alzira Batista Cecílio, Gustavo Portela Ferreira, Betânia Paiva Drumond, Jaquelline Germano de Oliveira, Cláudio Antônio Bonjardim, Paulo César Peregrino Ferreira, Erna Geessien Kroon

**Affiliations:** * Universidade Federal de Minas Gerais, Belo Horizonte, Minas Gerais, Brazil; † Fundação Ezequiel Dias, Belo Horizonte, Minas Gerais, Brazil

**Keywords:** Dengue virus, phylogeny, dengue type 3 virus genotype 1, dispatch

## Abstract

Dengue serotype 3 viruses were isolated from patients in Brazil from 2002 through 2004. On the basis of phylogenetic analyses, these isolates were assigned genotype 1. This genotype had never been reported in South America before. Its appearance indicates a major risk factor for dengue epidemics and severe disease.

Currently, dengue is the most significant mosquito-borne viral disease that affects humans. *Dengue virus* (DENV) is transmitted to humans by *Aedes aegypti* mosquitoes; for most persons, this infection is either asymptomatic or dengue fever (DF) develops. In a few cases, DF can progress to life-threatening dengue hemorrhagic fever/dengue shock syndrome (DHF/DSS). Epidemiologic and phylogenetic studies indicate that particular DENV strains are more virulent than others (*1**,**2*). DENV comprises 4 serotypes. Phylogenetic and molecular analyses showed extensive variability among the DENV serotypes, which led to the recognition of different genotypes within each serotype. For DENV-1 and DENV-2, five genotypes have been described (*2*); DENV-3 and DENV-4 have been subdivided into 4 and 2 genotypes, respectively (*3**,**4*). Regarding DENV-3, genotype 1 includes isolates from Southeast Asia and the South Pacific islands; genotype 2, Thailand; genotype 3, the Indian subcontinent, East Africa, and a single isolate from Samoa; and genotype 4, Puerto Rico and Tahiti (*3*).

Since 2000, DENV-1, DENV-2, and DENV-3 have been found cocirculating in 22 of the 27 states in Brazil (*5**,**6*). DENV-3 in Brazil belongs to genotype 3 and includes strains from Sri Lanka, India, and Africa (*6**–**8*). Since 1996, successive epidemics have been occurring in the city of Belo Horizonte (estimated population 2,214,000), which is located in the south-central region of Minas Gerais State, Brazil. Of the 700,000 dengue cases reported in Latin America in 1998, 12.4% were from Belo Horizonte, and 58.8% were from Minas Gerais State, respectively (*9*). Besides DENV-1 and DENV-2, the DENV-3 serotype was also detected in Minas Gerais State, but only a few DENV-3 isolates have been analyzed with respect to their genetic variability.

## The Study

**Figure 1:**
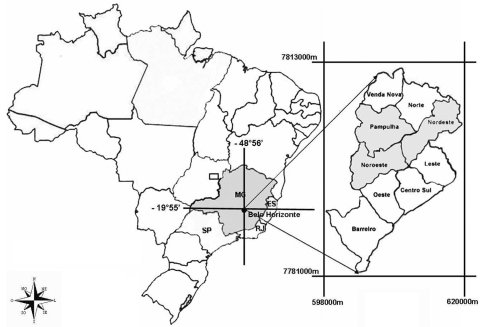
Location of the city of Belo Horizonte and its boroughs Pampulha, Nordeste, and Noroeste, Minas Gerais State, where samples of dengue virus serotype 3 were collected during 2002–2004.

We analyzed the C-prM and the envelope genes of DENV-3 isolates related to different clinical manifestations of dengue disease from Minas Gerais State, Brazil, from 2002 through 2004. Nine acute-phase serum samples from patients with DF or DHF (previously identified by PCR as DENV-3) were selected for this study (Table). All serum samples were from patients living in the city of Belo Horizonte or neighboring cities (Figure 1). Only 1 case-patient (patient MG-20) died.

**Table:** Description of the dengue virus serotype 3 isolates*

Identification	Disease	Patient sex	Patient age, y	City	Year	Passage history†	GenBank accession nos.	
							E sequence	C-prM sequence
BH–16	DF(HM)	M	42	Belo Horizonte	2003	5	EF625832	EF428575
BH–17	DF(HM)	M	6	Belo Horizonte	2003	4		EF428567
BH–19	DF	M	29	Belo Horizonte	2003	5	EF625833	EF428574
MG–20‡	DHF	F	18	Contagem	2004	4	EF625835	EF428572
MG–21	DF	F	37	Sabará	2003	5		EF428568
BH–22	DHF	F	11	Belo Horizonte	2004	5		EF428571
BH–24	DF	F	37	Belo Horizonte	2003	6	EF625834	EF428570
BH–25	DF	M	25	Belo Horizonte	2003	3		EF428569
MG–27	DF	–	–	Caetanopólis	2002	6		EF428573

*DF, dengue fever; DF(HM), dengue fever with hemorrhagic manifestations; DHF, dengue hemorrhagic fever, according to World Health Organization definition; –, data not available. †Number of passages in cell culture (C6/36 cell line) is shown. ‡Fatal case.

For viral isolation, 50 μL of each serum sample was incubated with C6/36 cells, and at least 3 successive passages were conducted for each sample. Microscopic examination of cells inoculated with serum from the patients showed a clearly visible cytopathic effect with changes in the monolayer such as syncytial cell formation and cytoplasmic vacuoles after the third passage (data not shown).

Supernatants of infected C6/36 cells showing typical cytopathic effect were used for viral RNA extraction (QIAamp Viral RNA Kit, QIAGEN, Inc., Valencia, CA, USA). RNA was used as template in reverse transcription–PCR (RT-PCR), as described (*10*).

For determination of nucleotide sequences in the C-prM region from 9 virus samples, the amplicons were cloned into a pGEM-T vector (Promega Corp., Madison, WI, USA), and 3 clones for each isolate were used in sequencing reactions. To determine the nucleotide sequence of the envelope gene from 4 isolates, we purified PCR amplicons (QIAquick Gel Extraction Kit, QIAGEN) and directly used in sequencing reactions. Each DNA sample was sequenced at least 3 times in both orientations (MegaBACE sequencer, GE Healthcare, Buckinghamshire, UK). Nucleotide sequences were aligned with other DENV-3 sequences. The midpoint rooted phylogenetic trees were constructed by the neighbor-joining method with 1,000 bootstrap replicates using the Tamura Nei model implemented by the software MEGA 3.1 (Arizona State University, Phoenix, AZ, USA).

**Figure 2:**
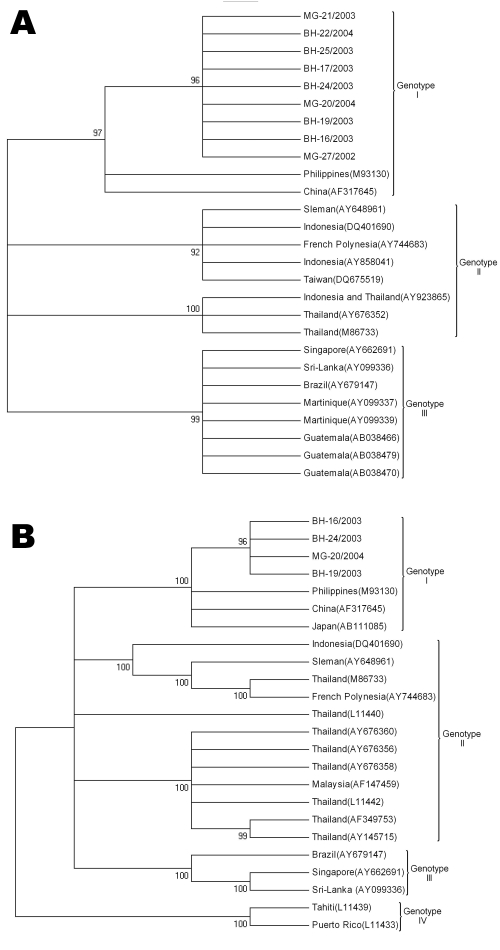
Phylogenetic trees of established dengue virus serotype 3 (DENV-3) and new sequences from Minas Gerais State, Brazil, identified in this study. A) The tree is based on a 504-nt sequence alignment comprising the C-prM gene (nucleotides 137–638). B) The tree is based on a 1,023-nt partial E nucleotide sequences (nucleotides 1022–2008). This tree was generated by neighbor-joining using the Tamura Nei model implemented by using MEGA3 software (www.megasoftware.net). Numbers to the left of nodes represent bootstrap values (1,000 replicates) in support of grouping to the right. Numbers to the right in parentheses of branches indicate the GenBank accession number. Roman numerals denote the different genotypes of DENV-3.

Sequence comparisons showed high degrees of identity among our isolates, and the paired identity at the nucleotide level ranged from 99.2% to 100% and from 99.7% to 99.9% regarding the C-prM region and the envelope gene, respectively. When sequences were compared with genotype 3 isolates, including isolates from Latin America, the Indian subcontinent, and East Africa, the identity values ranged from 95.4% to 96.2% and from 94.0 to 95.6% in relation to the C-prM and envelope genes, respectively. When those were compared to genotype 1 sequences from the Philippines and China, the nucleotide identities of C-prM region ranged from 98.4% to 99.4%; when envelope sequences were analyzed, values from 99.1% to 99.5% nucleotide identity were observed. According to phylogenetic clustering with other DENV strains (Figure 2), viruses were classified into a specific serotype and genotype. Both phylogenetic trees showed isolates from Brazil grouped together in a well-supported distinct cluster of genotype 1 isolates.

##  Conclusions

Various genomic regions of DENV have been used for molecular phylogenetic analyses. As described (*10**,**11*), the C-prM junction and envelope genes have been used as the most sensitive method for virus detection because they harbor epidemiologically relevant sequence information.

Although consensus nucleotide sequences of DENV isolated from different localities have provided some measure of genetic diversity, only a small number of studies use viruses isolated from the same location. Moreover, phylogenetic studies indicated an association between specific genotype and the severity of the disease (*8**,**12*).

By analyzing a conserved region of the DENV genome (504 nt of C-prM gene) and a more variable region (1,023 nt of the envelope gene), we verified that the DENV-3 isolates belong to genotype 1 (Figure 2). Other DENV-3 isolates sampled from Rio de Janeiro, Brazil, from 2001 to 2002 during an outbreak and also in Latin America were assigned to genotype 3, which has been associated with DHF outbreaks (*6**–**8**,**13*).

Phylogenetic studies have shown that DENV can move long distances between continents as well as short distances between neighboring countries (*8**,**14*). In this study, all DENV-3 isolates, which were associated with DF, DHF, and a fatal case, belonged to genotype 1. However, no consistent differences among the isolates in relation to C-prM or envelope sequences were found to be associated with distinct clinical outcomes nor did they form phylogenetically distinct groups. If the disease severity in DENV-3–infected patients does have a genetic basis, it cannot be attributed to the C-prM or envelope gene.

DENV-3 genotype 1 in Minas Gerais State is more likely to be imported from Asia because these isolates were closely related in the phylogenetic tree to the reference strain from the Philippines and China (*15*). It is difficult to determine the precise route of importation of DENV into Minas Gerais because the available data are limited. But one could speculate that the introduction of genotype 1 into Minas Gerais occurred in 2002, because this genotype had not been detected in that area before 2002. This study shows the emergence of a new DENV-3 genotype not only in Minas Gerais, but in the entire subcontinent, which is a matter for prospective public health studies.
